# Comprehensive immune modulation mechanisms of Angong Niuhuang Wan in ischemic stroke: Insights from mass cytometry analysis

**DOI:** 10.1111/cns.14849

**Published:** 2024-07-29

**Authors:** Yang Yao, Weihua Ni, Liangshu Feng, Jihong Meng, Xiaomu Tan, Hansen Chen, Jiangang Shen, Heng Zhao

**Affiliations:** ^1^ Department of Neurosurgery Stanford University School of Medicine Stanford California USA; ^2^ Department of Neurology Tianjin Medical University General Hospital Tianjin China; ^3^ School of Chinese Medicine, State Key Laboratory of Pharmaceutical Biotechnology The University of Hong Kong Hong Kong China; ^4^ Beijing Institute of Brain Disorders, Laboratory of Brain Disorders, Ministry of Science and Technology, Joint Innovation Center for Brain Disorders Capital Medical University Beijing China

**Keywords:** AGNHW, anti‐inflammation, immunomodulation, mass cytometry, neuroprotection, stroke

## Abstract

**Background:**

Angong Niuhuang Wan (AGNHW, 安宫牛黄丸), is a classical medicinal formula in Traditional Chinese Medicine (TCM) that has been appreciated for its neuroprotective properties in ischemic cerebral injuries, yet its intricate mechanisms remain only partially elucidated.

**Aims:**

This study leverages advanced Mass cytometry (CyTOF) to analyze AGNHW's multifaceted immunomodulation effects in‐depth, emphasizing previously underexplored areas.

**Results:**

AGNHW mitigated monocyte‐derived macrophages (MoDM) infiltration in the brain, distinguishing its effects on those from microglia. While the vehicle group exhibited elevated inflammatory markers like CD4, CD8a, and CD44 in ischemic brains, the AGNHW‐treated group attenuated their expressions, indicating AGNHW's potential to temper the post‐ischemic inflammatory response. Systemically, AGNHW modulated fundamental immune cell dynamics, notably augmenting CD8^+^ T cells, B cells, monocytes, and neutrophil counts in the peripheral blood under post‐stroke conditions. Intracellularly, AGNHW exhibited its targeted modulation of the signaling pathways, revealing a remarked inhibition of key markers like IκBα, indicating potential suppression of inflammatory responses in ischemic brain injuries.

**Conclusion:**

This study offers a comprehensive portrait of AGNHW's immunomodulation effects on ischemic stroke, illuminating its dual sites of action—both cerebral and systemic—and its nuanced modulation of cellular and molecular dynamics.

## Graphical abstract

By employing the advanced CyTOF technique, we conducted an in‐depth analysis to uncover the protective mechanisms of AGNHW. Leveraging the precision of CyTOF, our study provides a comprehensive understanding of AGNHW's therapeutic potential in ischemic stroke. It emphasizes its dual action, affecting both the cerebral and systemic aspects, and its nuanced modulation of cellular and molecular dynamics.

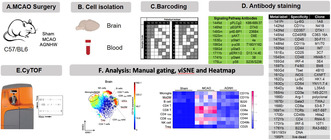

## 1 INTRODUCTION

Stroke remains a primary contributor to worldwide mortality and morbidity. Despite the availability of only two primary FDA‐endorsed treatments including intravenous thrombolysis and mechanical thrombectomy,^1^, ^2^ they cater to less than 15% of stroke victims.^3^ Although our understanding of its etiology has grown, therapeutic options remain narrow.

Traditional Chinese Medicine (TCM) offers remedies for stroke treatment. Angong Niuhuang Wan (AGNHW, 安宫牛黄丸), a classical TCM formulation, is accredited by China's State Food and Drug Administration for its detoxifying, revival, and anticonvulsant attributes, especially for conditions such as acute stroke, encephalitis, and meningitis.^4^, ^5^ Empirical assessments highlight AGNHW's protective role in ischemic stroke animal models, reducing infarct magnitude, mitigating brain swelling, and enhancing neurological performance.^6^, ^7^ Our previous studies indicate that AGNHW has neuroprotective effects in experimental ischemic stroke. Specifically, it protects the integrity of the blood–brain barrier (BBB), reduces brain edema, and improves neurological deficit scores. The underlying neuroprotective mechanisms are related to scavenging reactive nitrogen species (RNS) and reactive oxygen species (ROS), inhibiting MMP activity and decreasing neural cell death.^8^, ^9^ Significantly, our findings indicate that mineral constituents such as realgar and cinnabar play a crucial role as essential components contributing to the neuroprotective effects observed.^9^ In addition, our study revealed that AGNHW had a preventive effect on delayed t‐PA thrombolysis‐induced hemorrhagic transformation in an experimental ischemic stroke model. Furthermore, AGNHW treatment resulted in improved neurological function and reduced mortality rates.^10^ Our recent studies on ischemic stroke animal models demonstrate that a week‐long consistent oral administration of AGNHW is safe and beneficial for managing cerebral ischemia in vivo.^8^, ^9^ We previously reviewed the literature for 40 years (1975–2014) on the reported adverse drug reactions (ADR) and adverse events (AE) and demonstrated the safety of AGNHW with seldom ADR/AE reports in patients.^11^ Nonetheless, the intricate cellular and molecular pathways underscoring its protective attributes in the context of ischemic stroke remain opaque.

Ischemic stroke‐induced brain damage, characterized by reduced cerebral blood flow (CBF), initiates a cascade of molecular events that exacerbate tissue injury. This damage involves various pathological mechanisms, including cellular excitotoxicity, mitochondrial dysfunction, blood–brain barrier (BBB) impairment, neuroinflammation, and cell death processes.^12^, ^13^ While AGNHW's neuroprotective benefits in cerebral ailments are recognized, past explorations were often restricted in their breadth and depth.^6^, ^14^ In addition, the crucial roles of distinct immune entities, in both cerebral and peripheral blood spheres, largely remain untouched in AGNHW studies.

To address these shortcomings, we endeavor to revamp the AGNHW discourse by plumbing its therapeutic depths. With mass cytometry (CyTOF) at our disposal, we have transcended conventional boundaries.^15^ This advanced approach allows for detailed analysis of an extensive cellular indicator spectrum while sidestepping traditional spectral overlap challenges. By bridging the existing knowledge gaps and employing cutting‐edge analytical tools, this study aims to present a comprehensive and refined understanding of AGNHW's therapeutic potential, setting the stage for more targeted and effective interventions in ischemic stroke therapy.

## 2 MATERIALS AND METHODS

### 2.1 Animals

Male C57BL/6 mice were purchased from Jackson Laboratory (Sacramento, CA, USA), 8–10 weeks old, average weight of 22–25 g for this study. Random allocation was used to assign the mice to different experimental groups. The Institutional Animal Care and Use Committee of Stanford University approved all experimental procedures and protocols. This study was conducted in accordance with the guidelines outlined in the National Institutes of Health Guide for the Care and Use of Laboratory Animals (NIH; Bethesda, MD, USA) and followed the Animal Research: Reporting In Vivo Experiments guidelines.^16^, ^17^ The mice were housed in a specific‐pathogen‐free (SPF) environment at the Stanford University Veterinary Service Center. They had access to standard food and water ad libitum and were kept on a 12‐h light–dark cycle. The temperature in the facility was maintained between 65 and 75 °F, and the humidity level was kept within the range of 40%–60%. Each cage accommodated a maximum of five mice.

### 2.2 Middle cerebral artery occlusion (MCAO) model

The mice were induced with transient MCAO ischemia following the operational procedure as previously described.^18^ Anesthesia was induced using 5% isoflurane and subsequently maintained under anesthesia with 2% isoflurane in a gas mixture (70% N_2_O, 30% O_2_). Oxygen at 30% concentration is often used in animal stroke models to ensure sufficient oxygen delivery to the brain tissue.^19^, ^20^, ^21^ Throughout the surgical procedures, the core body temperature was maintained at 37 ± 0.5°C. An incision was made on the common carotid artery (CCA), and a 0.23 mm diameter tip nylon monofilament coated with silicone rubber (Doccol, CA, USA) was inserted into the right middle carotid artery (MCA) through the CCA incision. After a 45‐min occlusion of circulation, the suture was gently withdrawn. Collar sutures around the CCA were tightened to ensure permanent ligation. Reperfusion of the MCA occurred after filament removal due to the redundant blood supply through the robust circle of Willis. CBF was continuously recorded using a laser Doppler flowmetry (Moor Instruments, Wilmington, DE, USA) including a duration of 5 min before and after MCAO, as well as the initial 10 min of reperfusion (Figure S2). The mice that exhibited less than 80% CBF reduction were excluded. The ratio of mice with successful reperfusion after stroke was more than 90%. The mice in the sham group underwent a similar surgical procedure without MCA occlusion.

### 2.3 Drug administration

AGNHW was provided by the Beijing Tong Ren Tang Chinese Medicine Company Limited (Hong Kong, China). The AGNHW was dissolved in saline, and the mice received AGNHW via oral gavage at a dosage of 257 mg/kg at three time points (2, 24, and 48 h after reperfusion). As previously reported,^22^ the dosage given to the mice was determined to be equivalent to the daily dosage used in human subjects, which is 3 g per pill per day. The vehicle control mice received an equal volume of saline.

### 2.4 Neurological function assessment

All the assessments were carried out by investigators blinded to each group. The assessment of comprehensive neurological functions, including motor, sensory, reflex, and balance was conducted using modified neurological severity score (mNSS). After the surgery, the mNSS assessment was performed on days 1, 2, and 3, based on previously described methods.^23^ Sensorimotor and postural asymmetries were evaluated by the corner‐turning test.^24^ Vibrissae Evoked Forelimb placing test were conducted to assess the ability of mice to respond to a vibrissae‐elicited excitation.^25^ These mice were allowed 10 consecutive trials on each side, and the percentage of successful placements was calculated.

### 2.5 Assessment of infarct volume

After the neurological function scoring, the mice were euthanized using isoflurane at 3 days after MCAO. After perfusion with phosphate‐buffered saline (PBS), the whole brains were immediately removed and cut into 1 mm coronal sections. The sections were incubated in a 2% (w/v) solution of 2,3,5‐triphenyl tetrazolium chloride (TTC) (Sigma‐Aldrich, MO, USA) at 37°C for 20 min. After staining, the sections were fixed in 4% paraformaldehyde for 30 min at 4°C before being photographed. The infarct volumes were calculated by integrating the infarcted areas on each brain section using Image J software (National Institutes of Health, USA) as described previously.^26^, ^27^

### 2.6 Evans blue assay

Evans blue (EB) dye was used to assess vascular permeability in animal models, and its extravasation quantitation was used to analyze BBB permeability. Briefly, 3 days after reperfusion, 2% EBD (Sigma‐Aldrich, MO, USA) was intravenously administered to the mice. Two hours later, the mice were perfused and sacrificed. The hemisphere was weighed and subsequently homogenized with 2 mL of *N*, *N*‐Dimethylformamide (Sigma‐Aldrich, MO, USA). The homogenized samples were then incubated in a water bath at 60°C for 72 h. After incubation, the samples were centrifuged, and the absorption of the supernatant was read with a spectrophotometer at 620 nm. The EB concentration was expressed using the following formula: EB content (μg/mg) = EB concentration (μg/mL) × formamide (mL)/wet weight (mg).^28^

### 2.7 Mass cytometry

#### 2.7.1 Specimen collection and processing

The mice were euthanized on the third day after MCAO. Peripheral blood specimens were collected from the caudal vein in ethylenediaminetetraacetic acid anticoagulation (EDTA) tubes. The ischemic hemisphere was dissected and separated using Percoll density gradient centrifugation to obtain peripheral blood mononuclear cells (PBMCs). This approach allows us to evaluate changes not only in the necrotic core but also in the penumbra, ensuring a comprehensive understanding of the brain's inflammatory processes, as previously described.^29^, ^30^ Single cells were then washed, centrifuged, and resuspended in a staining buffer, following the previously described protocol.^29^

#### 2.7.2 CyTOF profiling

A previously reported panel of 35 antibodies was utilized in the study,^29^ all of which were obtained from Fluidigm Company and used in accordance with the manufacturer's user guide. To distinguish live and dead cells, we incubated the cell specimens with Cisplatin (Fluidigm, CA, USA) for 10 min. Following washing and fixation, the samples were barcoded. To block Fc‐receptor, a Fc receptor blocking solution (BioLegend, CA, USA) was added to the samples, followed by incubation for 10 min. After blocking, the samples were incubated with a mixed solution of antibodies (as specified in Table S2) in a final volume of 100 μL for 30 min at 23°C. The stained cells were incubated with 500 μL intercalating Iridium solution (Fluidigm, CA, USA) overnight at 4°C. Before running on CyTOF, the samples were washed twice with Maxpar cell staining buffer and resuspended with deionized water. Finally, EQ Calibration beads (Fluidigm, CA, USA) were added to the sample at a concentration of 2.5–5 × 10^5^/mL. The samples were processed using CyTOF2 (Fluidigm, CA, USA) with 300–500 events per second.^29^

#### 2.7.3 CyTOF data analysis

The acquired CyTOF data were normalized and debarcoded using the Helios software. Subsequently, Cytobank (www.cytobank.org) was utilized for further data processing and analysis. Manual gating, as previously described,^29^ was performed to identify specific immune cell populations of interest in individual samples. To visualize the data, we employed the viSNE which was implemented in Cytobank. The heatmap of relative marker expression was generated in GraphPad (San Diego, CA, USA). The ANOVA assay was used to compare the expression differences of immune signaling mediators between the vehicle and Sham groups in each cell type. For the comparison, functional proteins showing significant twofold differences (*p* < 0.01) between the MCAO and AGNHW groups were considered as AGNHW responding immune signaling mediators. The expression changes were analyzed using hierarchical clustering by distance, which was performed using the R package, as described previously.^29^

### 2.8 Real‐time PCR

TRIzol reagent (Invitrogen, CA, USA) was used to isolate total mRNA from ischemic hemisphere samples. RNA concentration was quantified by using a NanoDrop (Thermo Fisher Scientific, USA) at 260/280 nm. The PrimeScript™ RT reagent kit (Takara Bio, CA, USA) was used to transcribe RNA into cDNA according to the manufacture's protocol. Real‐time PCR was conducted using the Stratagene® MX300P system (Agilent Technologies, CA, USA). The amplification of gene sequences was performed using SYBR Green PCR Master Mix (Roche Diagnostics, IN, USA). GAPDH served as the reference gene. The expression levels of the mRNAs are presented as fold changes compared to the control. Detailed information on the primer sequences can be found in Table S1.

### 2.9 Western Blot

The ischemic hemisphere samples were harvested 3 days after MCAO and gently homogenized in RIPA buffer (Sigma‐Aldrich, MO, USA). The protein concentration was determined using the bicinchoninic acid protein detection system (Bio‐Rad, CA, USA). Proteins were separated by SDS‐PAGE and subjected to Western blotting using standard protocols. The following antibodies were used: anti‐ZO‐1, anti‐claudin‐5, and anti‐MMP9 (Cell Signaling Technology, MA, USA). Anti‐GAPDH was used as an internal control. Signals were detected using an ECL reagent (Amersham, NJ, USA), and densitometry analysis was performed using Image J software (National Institutes of Health, USA).

### 2.10 Statistical analysis

All data analysis was conducted by investigators who were blinded to the treatment conditions. Data are presented as the means ± SEM. The datasets were initially subjected to the Kolmogorov–Smirnov normality test and Shapiro–Wilk test, and all data were found to be normally distributed. Statistical significance was assessed using the following tests: Two‐tailed unpaired Student's *t* test was used for two groups, one‐way ANOVA followed by Tukey post hoc test was used for three groups, and two‐way ANOVA followed by Bonferroni post hoc test was used for multiple comparisons. GraphPad Prism 8.0 software (San Diego, CA, USA) was used for all statistical analyses and a statistically significant difference was set at *p*‐value less than 0.05.

## 3 RESULTS

### 3.1 AGNHW mitigates ischemic damage and bolsters neurological recovery

**FIGURE 1:**
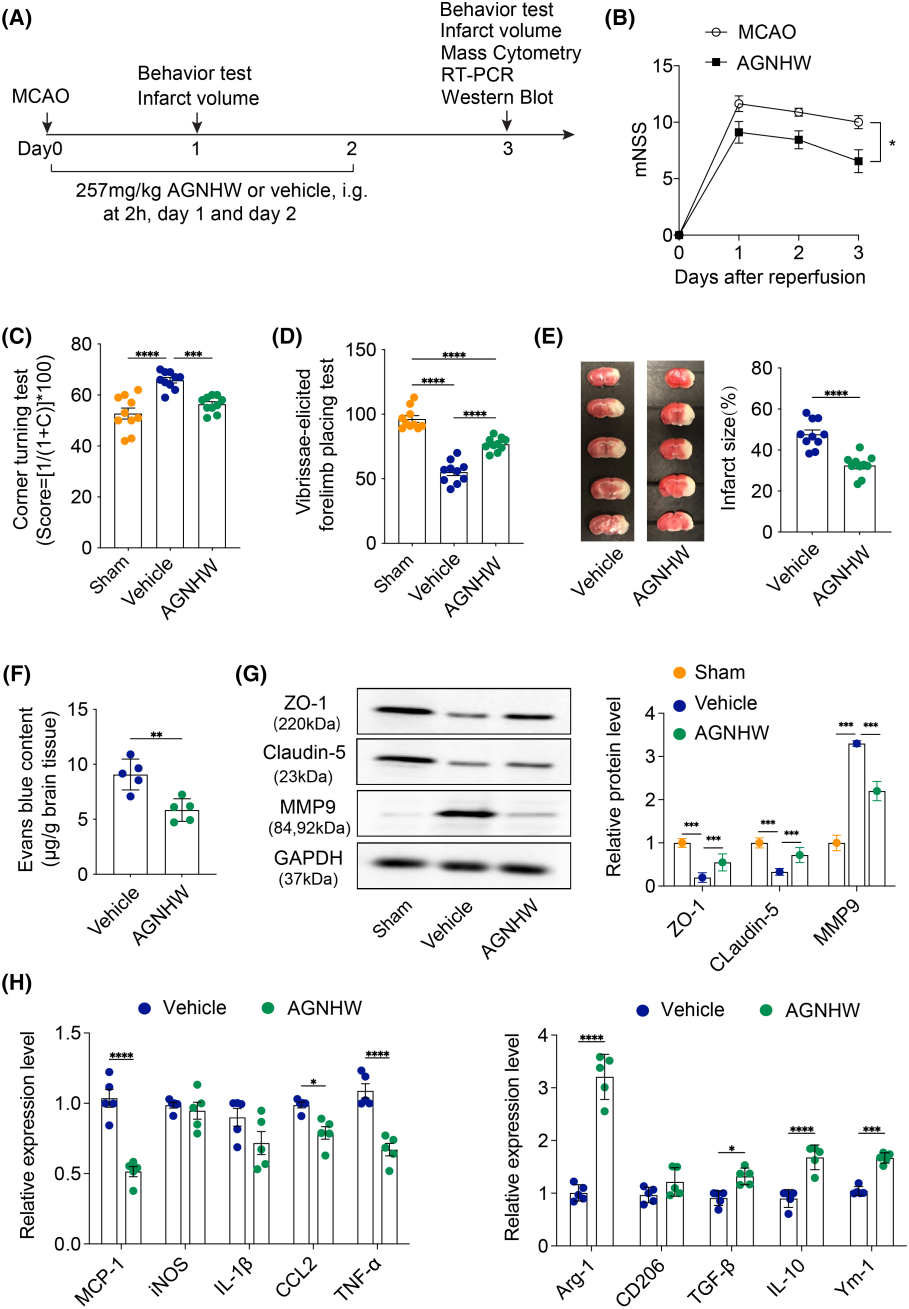
AGNHW treatment ameliorates neurological deficits and brain infarction after cerebral ischemia and reperfusion. (A) To assess the effects of AGNHW on neurological function and infarct volume in mice subjected to 45‐min middle cerebral artery occlusion (MCAO), an experimental scheme was implemented. AGNHW (257 mg/kg) or vehicle (saline) was administered orally via gavage 2 h after reperfusion and repeated daily for two consecutive days. Neurological deficits were evaluated using the modified neurological severity score (mNSS), corner turning test, and forelimb placing test. Infarct volume was quantified at 3 days using TTC staining. (B–D) The summarized results of the mNSS scores, corner turning test, and forelimb placing test were analyzed for mice receiving AGNHW or vehicle at different time points after reperfusion. The data, expressed as mean ± SEM, revealed statistically significant differences (*p < 0.05, **p < 0.01, ***p < 0.001) using two‐way ANOVA, *n* = 10 per group. (E) TTC‐stained brain slices displayed the infarct areas (white) in mice receiving AGNHW or vehicle at 3 days after reperfusion. The representative images showed significant differences (***p < 0.001) between the two groups, as determined by a two‐tailed unpaired Student's *t*‐test. These findings were consistent across three independent experiments, *n* = 10 per group. (F) The content of Evans blue was measured 3 days after ischemia stroke onset. The data, expressed as mean ± SEM, revealed statistically significant differences (**p < 0.01) between the two groups, *n* = 5 per group (G) Western blot analysis demonstrated the expression of MMP‐9, ZO‐1, and Claudin‐5. The data, presented as mean ± SEM, exhibited statistically significant differences (*p < 0.05, **p < 0.01, ***p < 0.001) between the two indicated groups, with a sample size of five per group. (H) qRT‐PCR for the mRNA expression of M1 (MCP‐1, iNOS, IL‐1β, CCL‐2, TNF‐α) and M2 (Arg‐1, CD206, TGF‐β, IL‐10, and YM‐1) cytokines in the ischemic hemispheres in the AGNHW treatment group and vehicle group (*n* = 5 per group). *p < 0.05, **p < 0.01, ***p < 0.001, respectively, between the two indicated groups.

Following a 45‐min MCAO procedure, the MCAO mice were administered either AGNHW (257 mg/kg) or a vehicle solution orally at 2 h post‐reperfusion, with the treatment continuing daily for two subsequent days (Figure 1A). Our observations revealed a pronounced enhancement in neurological function and a notable reduction in infarct volume in AGNHW‐treated mice compared to those receiving the vehicle treatment (Figure 1B–E). Diving deeper into AGNHW's protective mechanisms, we explored its role in upholding the integrity of the BBB. This was evident from a diminished extravasation of Evans blue in the AGNHW group (Figure 1F). The results were consistent with our previous study.^10^ Furthermore, concerted retention in the expression of tight junction proteins, ZO‐1, and claudin‐5, was observed, alongside a concomitant decrease in MMP‐9 expression, underscoring AGNHW's protective stance (Figure 1G). In an intriguing turn, we also discerned that AGNHW treatment skewed the microglia/macrophage polarization more toward the reparative M2 phenotype. This was substantiated by the suppressed expression of M1‐associated gene markers and an amplified expression of M2‐associated ones (Figure 1H). Collectively, our data corroborate the notion that AGNHW confers neuroprotection in the context of ischemic cerebral insult, plausibly orchestrated through a myriad of mechanistic pathways.

### 3.2 AGNHW reveals immune cell modulation in ischemic brain and peripheral blood

Our objective was to investigate the impact of AGNHW on the composition of immune cells following ischemic insult. To achieve this, we employed CyTOF for comprehensive immune cell profiling (Figure S1).

**FIGURE 2:**
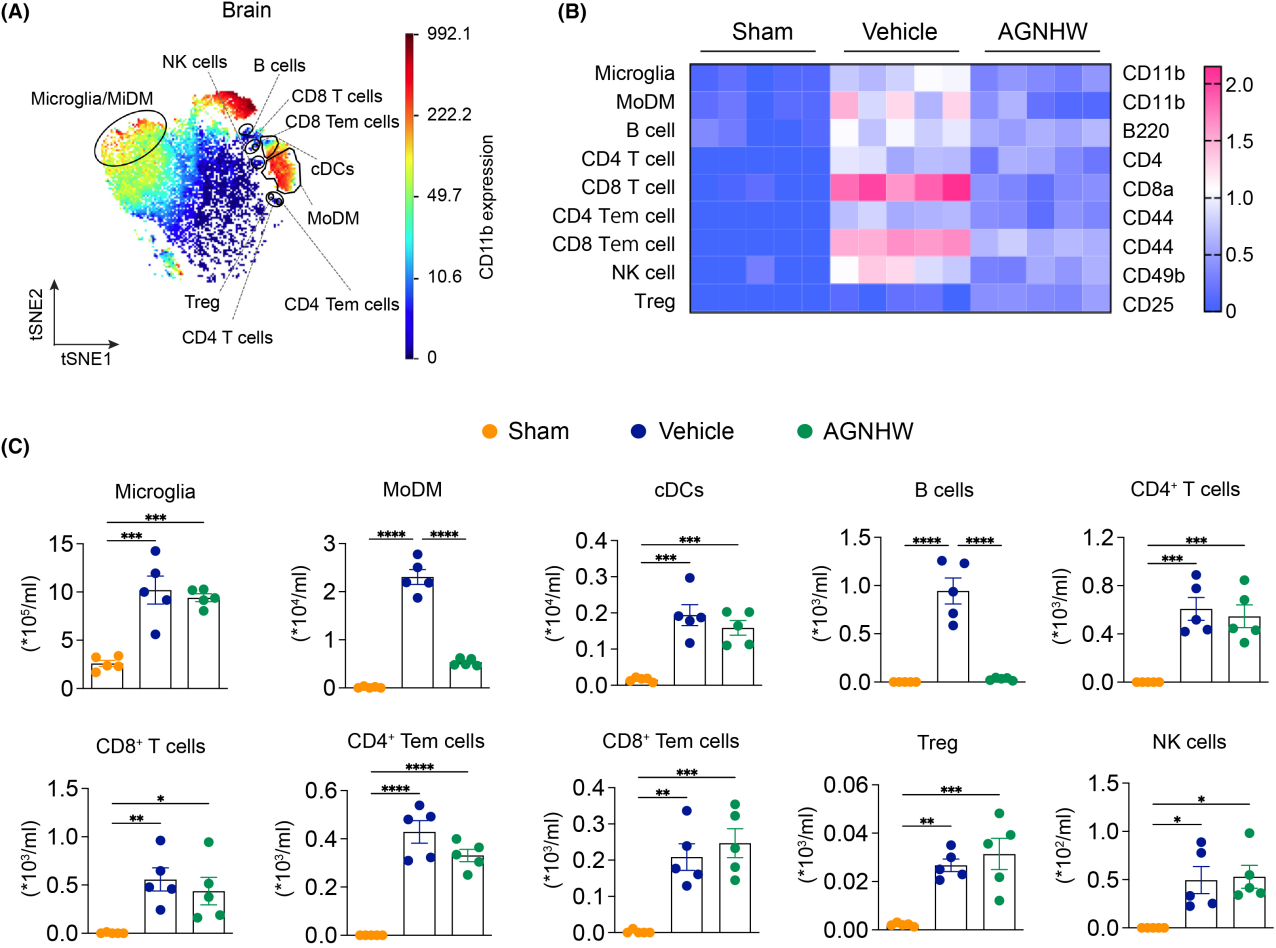
Mass cytometry reveals immune cell subsets in ischemic brain at day 3 after AGNHW treatment. (A) viSNE plots of clustered CD45^+^ cells were generated in representative mice at 3 days after ischemia in the ischemic brain. The analysis revealed the identification of 10 distinct populations, including myeloid, lymphocytes, and unknown subsets. (B) A heatmap was constructed to display the arcsinh ratio of median values, with the minimum value of each protein expression serving as the control. The expression levels of various cell surface markers were analyzed, including CD11b on microglia and monocyte‐derived macrophages (MoDMs), B220 on B cells, CD4 on CD4^+^ T cells, CD8 on CD8^+^ T cells, CD44 on CD4 effector memory T cells (Tem) and CD8 Tem cells, CD25 on regulatory T cells (Tregs), CD11c on conventional dendritic cells (cDCs), and CD49b on natural killer (NK) cells. (C) The quantification of immune cell subsets identified in the viSNE clustering was performed for the sham, vehicle, and AGNHW‐treated groups. The data, presented as mean ± SEM, displayed individual samples as dots on the bars. A statistical analysis revealed significant differences (*p < 0.05, **p < 0.01, ***p < 0.001) between the indicated groups. The sample size for each group was 5.

The viSNE plot visualized CD45^+^ clustering in the ischemic brains of mice at day 3 post‐stroke (Figure 2). Ten immune cell populations were identified, including microglia, MoDM, B cells, CD4^+^ T cells, CD8^+^ T cells, CD4 Tem and CD8 Tem, Tregs, cDCs, and NK cells (Figure 2A). A heatmap displayed the relative expression levels of markers in sham, vehicle, and AGNHW treatment groups, using color gradients to represent the expression levels (Figure 2B). The heatmap shows that vehicle treatment group had significantly increased expression of CD11b (microglia and MoDMs), B220 (B cells), CD4 (CD4^+^ T cells), CD8a (CD8^+^ T cells), CD44 (CD4 Tem and CD8 Tem cells), CD25 (Tregs), CD11c (cDCs), and CD49b (NK cells), indicating an elevated immune response and inflammation following ischemic stroke. In contrast, AGNHW treatment reduced the expression of these markers compared to the vehicle group, suggesting its potential to modulate the immune response and mitigate inflammation in the ischemic brains. We also quantified immune cell types in the ischemic brains (Figure 2C). Specifically, AGNHW treatment resulted in relatively low counts of MoDM and B cells.

**FIGURE 3:**
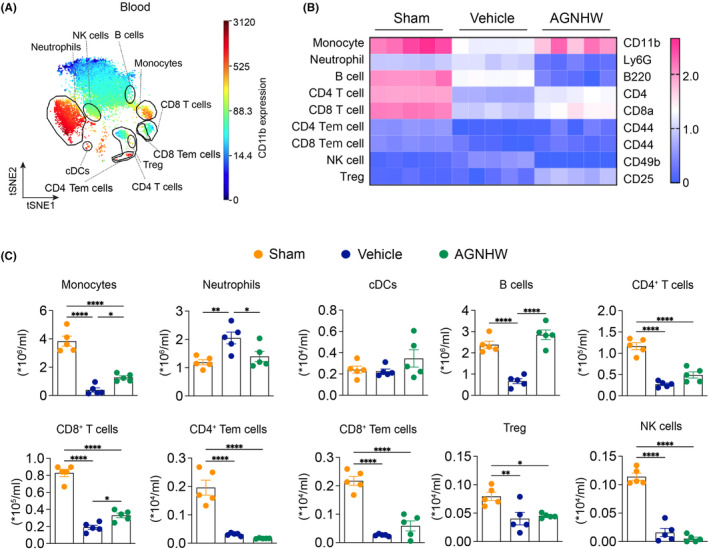
Mass cytometry reveals immune cell subsets in peripheral blood at day 3 after AGNHW treatment. (A) viSNE plots of clustered CD45^+^ cells were generated in representative mice at 3 days after ischemia in the peripheral blood. The analysis revealed the identification of 10 distinct populations, including myeloid, lymphocytes, and unknown subsets. (B) A heatmap was constructed to display the arcsinh ratio of median values, with the minimum value of each protein expression serving as the control. The expression levels of various cell surface markers were analyzed, including CD11b on monocytes, B220 on B cells, CD4 on CD4^+^ T cells, CD8 on CD8^+^ T cells, CD44 on CD4 Tem and CD8 Tem cells, CD25 on Tregs, CD11c on cDCs, and CD49b on NK cells. (C) The quantification of immune cell subsets identified in the viSNE clustering was performed for the sham, vehicle, and AGNHW‐treated groups. The data, presented as mean ± SEM, displayed individual samples as dots on the bars. A statistical analysis revealed significant differences (*p < 0.05, **p < 0.01, ***p < 0.001) between the indicated groups. The sample size for each group was 5.

In the peripheral blood (Figure 3), the viSNE plots show distinct clustering of 10 immune cell subsets, including monocytes, neutrophils, B cells, CD4^+^ T cells, CD8^+^ T cells, CD4 Tem and CD8 Tem, Tregs, cDCs, and NK cells (Figure 3A). The heatmap illustrates the changes in the expression levels of key cell surface markers across different treatment groups. In the vehicle‐treated group, there was a notable reduction in the expression of several markers compared to the sham group (Figure 3B). Specifically, CD11b (monocytes), B220 (B cells), CD4 (CD4^+^ T cells), and CD8a (CD8^+^ T cells) were significantly downregulated, which were reversed by AGNHW treatment. Also, AGNHW treatment effectively restored the levels of monocytes, B cells, and CD8^+^ T cells compared to the vehicle group, suggesting restorative effects on these immune cells. AGNHW also reduced neutrophil counts (Figure 3C).

### 3.3 AGNHW modulates immune responses in post‐ischemic brain injury through targeted immunological signaling pathways

We further investigated its impact on intracellular immune signaling dynamics within the ischemic brains and peripheral blood. Employing advanced bioinformatics techniques, we scrutinized the expression and phosphorylation profiles of immune signaling mediators.

**FIGURE 4:**
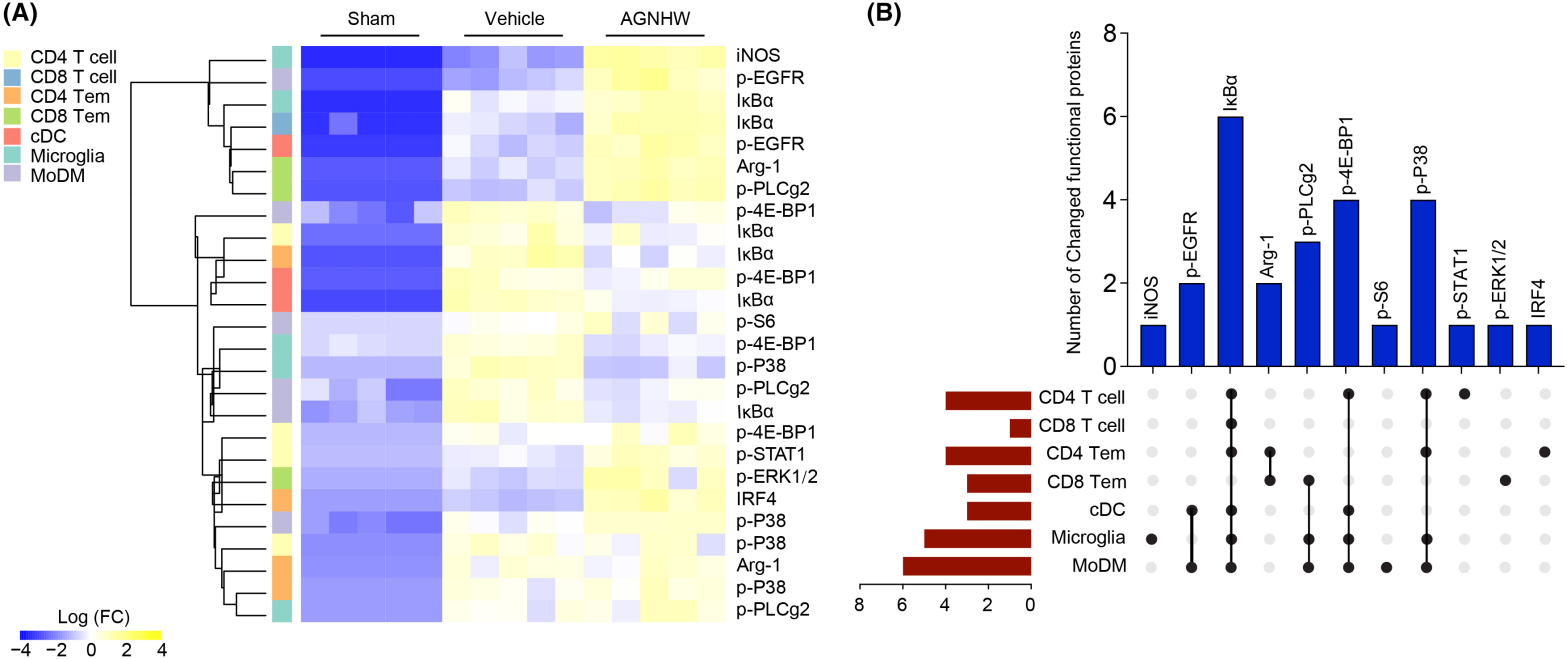
Immune signaling mediators' responses to AGNHW treatment in ischemic brain. (A) The heatmap presented significant changes in immune signaling mediators across different immune cell populations at day 3 after ischemia in the ischemic brain. The expression changes were normalized to the mean value of the sham group and displayed using hierarchical clustering by distance, implemented using the R package. Immune signaling mediators that exhibited significant differences (at least twofold change, adjusted *p*‐value <0.01) between the vehicle and AGNHW‐treated groups were selected. (B) The UpSet plot illustrates the number of immune signaling mediators that have undergone changes in different subsets of immune cells. The black vertical bars represent the count of changed immune signaling mediators. The dots positioned below the black bars indicate the specific immune cell types within the ischemic brain at day 3 after AGNHW treatment that exhibit alterations in immune signaling mediators. The red horizontal bars indicate the number of immune signaling mediators with significant changes across various immune cell populations. The sample size for each group was 5.

For the ischemic brain results, the heatmap illustrates significant changes in the expression of immune signaling mediators within various immune cell populations in the ischemic brain at day 3 post‐ischemia, particularly between the vehicle and AGNHW‐treated groups (Figure 4A). Key proteins such as iNOS, IκBα, p‐EGFR, and Arg‐1 exhibited notable alterations, indicating AGNHW's impact on critical inflammation and cell survival signaling pathways. The heatmap displayed a wide range of protein expression changes, with color intensity representing the degree of change (Log FC). In addition, the UpSet plot quantified the number of immune signaling mediators that underwent significant changes across different immune cell subsets following AGNHW treatment (Figure 4B). Proteins like p‐EGFR, iNOS, and Arg‐1 showed changes across multiple cell types, with vertical bars indicating the count of proteins with substantial alterations. The red horizontal bars showed the distribution and extent of these changes among various immune cell subsets such as CD4 T cells, CD8 T cells, microglia, and MoDMs. Notably, certain proteins like IκBα had the highest number of changes across the subsets, suggesting key roles in the response to AGNHW treatment.

**FIGURE 5:**
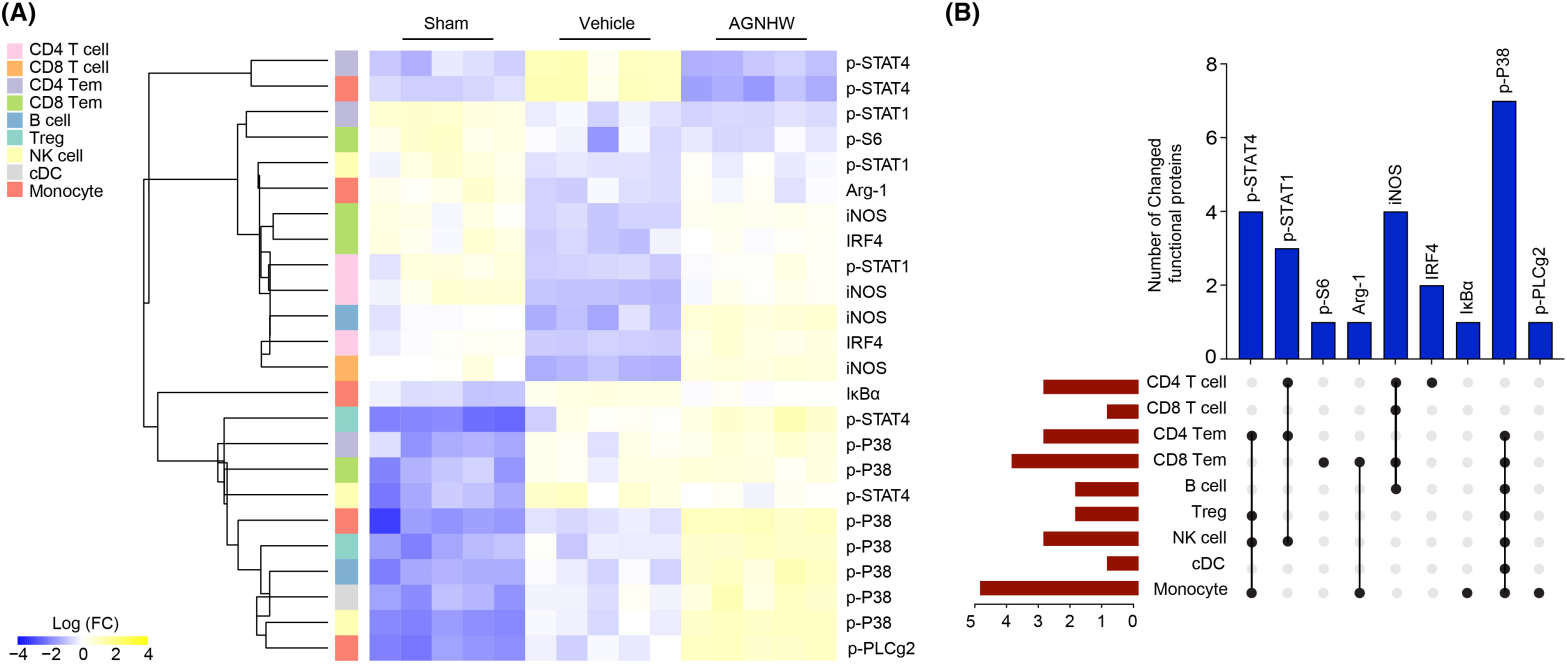
Immune signaling mediators' responses to AGNHW treatment in peripheral blood. (A) The heatmap presented significant changes in immune signaling mediators across different immune cell populations at day 3 after ischemia in the peripheral blood. The expression changes were normalized to the mean value of the sham group and displayed using hierarchical clustering by distance, implemented using the R package. Immune signaling mediators that exhibited significant differences (at least twofold change, adjusted *p*‐value <0.01) between the vehicle and AGNHW‐treated groups were selected. (B) The UpSet plot illustrates the number of immune signaling mediators that have undergone changes in different subsets of immune cells. The black vertical bars represent the count of changed immune signaling mediators. The dots positioned below the black bars indicate the specific immune cell types within the peripheral blood at day 3 after AGNHW treatment that exhibit alterations in immune signaling mediators. The red horizontal bars indicate the number of immune signaling mediators with significant changes across various immune cell populations. The sample size for each group was 5.

For the peripheral blood (Figure 5), the heatmap shows varying levels of expression changes for proteins like iNOS, IRF4, and several phosphorylated forms of STAT1, STAT4, and p38, which are key signaling molecules in immune responses. Notably, we observed significant changes in proteins such as p‐STAT4 and p‐STAT1 across cell types like CD4 T cells, CD8 T cells, NK cells, and monocytes. Monocytes were identified as the cell types with the most notable changes in immune signaling mediators in peripheral blood.

**FIGURE 6:**
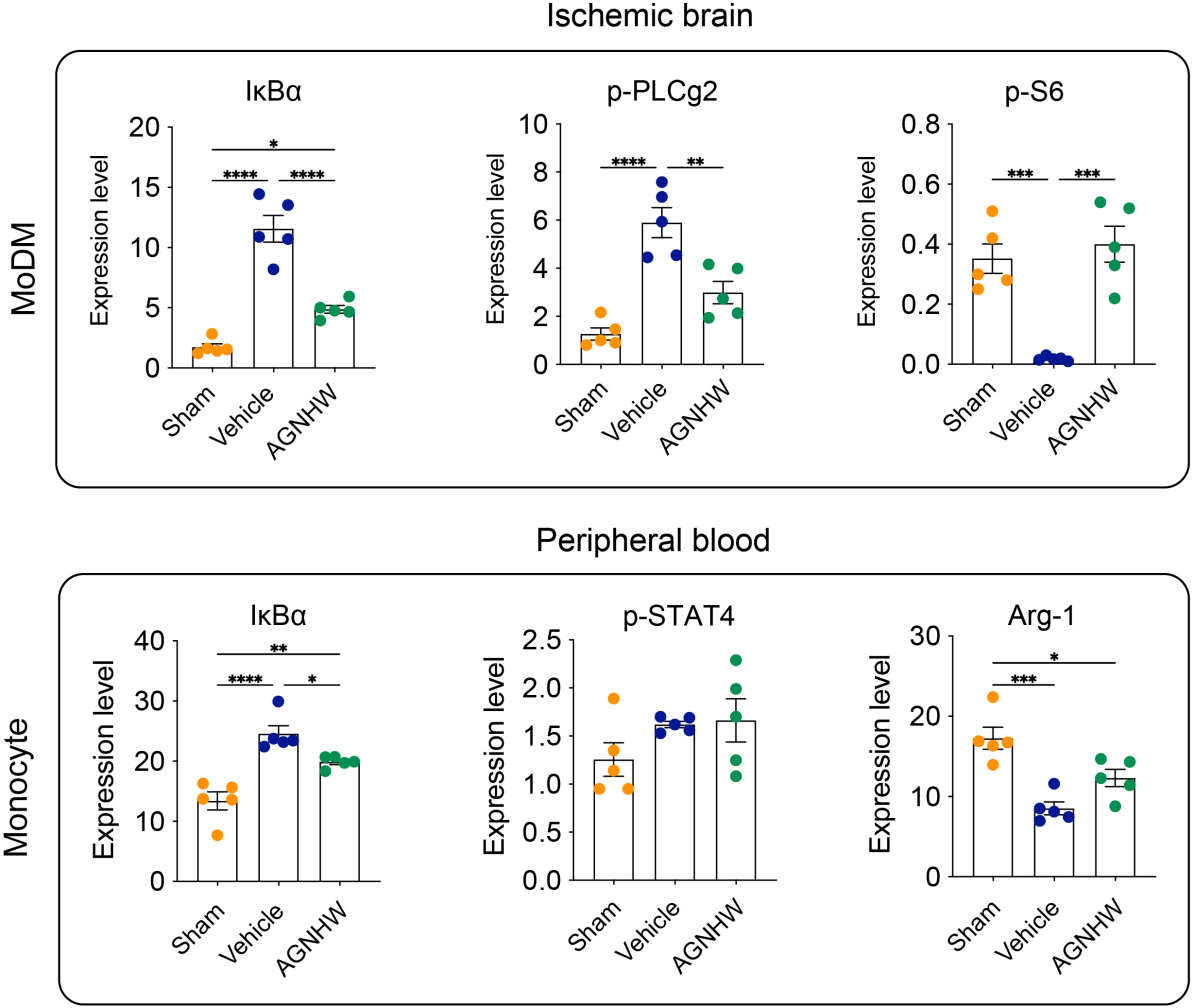
The expression of key immune signaling mediators in ischemic brain and peripheral blood. Bar graphs display the expression levels of IκBα, p‐PLCg2, and p‐S6 in MoDM. Additionally, the expression levels of IκBα, p‐STAT4, and Arg‐1 in monocyte were measured. Statistical significance was denoted as *p < 0.05, **p < 0.01, and ***p < 0.001, indicating differences between the two indicated group, *n* = 5 per group.

Furthermore, our findings demonstrated a significant reduction in the expression levels of IκBα, p‐PLCg2, and p‐S6 in MoDMs within the ischemic brains post‐treatment. Concurrently, analyses of peripheral blood highlighted a significant attenuation of IκBα expression in monocytes (Figure 6). Collectively, these observations suggest that the downregulation of IκBα could be one of the underlying mechanisms responsible for AGNHW's neuroprotective effects.

## 4 DISCUSSION

We demonstrated the therapeutic effects of AGNHW on mitigating the deleterious consequences of ischemic cerebral injuries, revealing the enhanced neurological outcomes, the minimized infarct size, and the bolstered BBB integrity as well as the protecting tight junction proteins and downregulating the expression of MMP‐9. Meanwhile, AGNHW redirected microglia/macrophage polarization from a proinflammatory M1 to an anti‐inflammatory M2 phenotype. Those results suggest the anti‐inflammatory effects might contribute to the neuroprotection of AGNHW against ischemic brain injury.

In our research, deploying mass cytometry (CyTOF) as an analytical tool provided a distinctive edge. Unlike conventional flow cytometry, CyTOF facilitated the simultaneous measurement of a more extensive array of cellular markers at the single‐cell level without the challenges of spectral overlap.^31^ This comprehensive profiling capability enabled us to dissect the intricate cellular responses and signaling pathways influenced by AGNHW with unparalleled resolution and depth. The use of this cutting‐edge technique not only ensured the rigor and precision of our findings and positioned our study at the forefront of ischemic stroke research employing advanced cellular analyses.

Stroke induces inflammation in the ischemic brain and reduces immune cells in peripheral blood, indicating immune suppression.^32^ Our study showed impacts of lymphopenia, decreased inflammatory cytokines, impaired monocyte and lymphocyte function, and atrophy of secondary lymphoid organs on ischemic brain injury. AGNHW reduced MoDM presence and immune signaling in ischemic brains and increased vital immune cells (CD8^+^ T cells, B cells, monocytes) in peripheral blood while reducing blood neutrophils. Ischemic stroke affects both innate and adaptive immune responses, triggering immediate immune activation, cytokine release, and immune cell recruitment to the injury site.^33^, ^34^ This response is crucial for clearing damaged tissues and initiating healing. AGNHW's ability to counter post‐stroke immune depression suggests a holistic approach to reduce inflammation and enhance recovery. Dynamic changes in immune cell numbers and immune signaling mediators could be targets of AGNHW treatment.

Historically, research on stroke‐induced neuroinflammation has primarily centered on microglia, the resident immune cells of the central nervous system, given their immediate response to cerebral injury and their significant role in modulating inflammation.^35^, ^36^ MoDMs, though equally integral to the immune response, have garnered comparatively less attention in the context of stroke. This underrepresentation might be attributed to the overlapping functions and phenotypes of MoDMs and microglia, making their distinct roles challenging to elucidate.^37^ However, emerging evidence, including findings from our research and studies conducted by other groups,^38^, ^39^ is shifting this narrative. There is a growing acknowledgment of the pivotal role MoDMs play in stroke‐induced brain injury, often distinct from that of microglia.^40^ In the present study, this distinction became prominently evident. While both cell types contribute to the post‐stroke inflammatory milieu, our results demonstrate that AGNHW specifically attenuated the activity and infiltration of MoDMs without significantly impacting microglia. This observation underscores the necessity to deepen our understanding of MoDMs in the context of stroke. It reinforces the potential of targeted therapies, such as AGNHW, that selectively modulate specific immune cell populations to foster neuroprotection.

Notably, following AGNHW treatment, significant alterations were observed in B cells and MoDM cells within the brains. AGNHW also affected B220 expression in peripheral blood B cells. B cells exhibit dual effects in ischemic injury. Some studies show their detrimental impacts, such as increased susceptibility to post‐stroke infections due to loss of splenic marginal zone B cells, affecting long‐term recovery.^41^, ^42^ Conversely, IL‐10‐secreting Bregs support recovery by offering acute neuroprotection and enhancing neurogenesis.^43^ Recent research identified age‐associated B cells that modulate microglial responses in the tMCAo model.^44^ Additionally, B220^low^ cells emerge post‐stroke and may produce autoantibodies.^45^ Given B cells' complex role in post‐stroke immune responses and changes in B220 expression, future studies should investigate their mechanisms and therapeutic potential following AGNHW treatment.

Evaluating cellular marker intensities post‐stroke offers crucial insights into immune responses following cerebral injury,^46^, ^47^ but few studies have explored these in depth. This gap may limit understanding of cell activation states and interactions post‐stroke. Our study highlighted a pronounced inflammatory response in the vehicle group, evidenced by elevated markers like CD4, CD8a, and CD44. CD8a is a potential prognostic and diagnostic marker for inflammatory disorders and tumors,^48^, ^49^ used to predict severity in chronic rhinosinusitis and as a biomarker for rheumatoid arthritis.^50^ CD8a expression is downregulated in a model of bronchopulmonary dysplasia.^51^ CD44, an adhesion molecule, is crucial in multiple cells and organs, with increased expression in microglia/macrophages following brain injuries, including ischemic stroke,^52^, ^53^ influenced by various cytokines and molecules.^54^ The AGNHW‐treated cohort showed attenuated expressions of these markers, suggesting its capacity to modulate the inflammatory milieu post‐ischemic insult, highlighting AGNHW's potential to balance inflammation while preserving essential immune functions.

Our study provides valuable insights into the neuroprotective effects of AGNHW following post‐ischemic insult. The reduction in IκBα, p‐PLCg2, and p‐S6 expression within MoDMs of the ischemic brain suggests targeted modulation of inflammation and cellular stress pathways. IκBα, an inhibitor of the NF‐κB signaling pathway, is downregulated by AGNHW, implying suppression of NF‐κB‐mediated inflammatory responses, which exacerbate ischemic injuries. Additionally, attenuated IκBα expression in peripheral monocytes of AGNHW‐treated rats highlights its systemic immunomodulatory effects. These findings illuminate AGNHW's therapeutic potential, operating via multiple signaling pathways to protect against ischemic brain injury. Future studies could use pathway blockade to further investigate the underlying mechanisms.

In this study, we analyzed the entire ischemic hemisphere to assess the inflammatory changes in both the necrotic core and penumbra, capturing their interplay.^55^ This comprehensive approach identifies therapeutic targets to protect the penumbra and mitigate secondary injury like oxidative stress and apoptosis.

We recognize several limitations in our current study. Our study had a specific focus on young male adult animals, which may limit the generalizability of our findings. Both age and gender are crucial factors that strongly influence stroke outcomes and neuroinflammatory responses.^56^, ^57^, ^58^ In addition, our MCAO model may not fully represent the dynamics of reperfusion that newer methods achieve. Nevertheless, even if the ipsilateral cervical carotid circulation is permanently occluded, reperfusion of the MCA occurs after filament removal due to the redundant blood supply through the robust circle of Willis.^59^, ^60^ Future investigations should include both female and aged animals and employ advanced MCAO models that reflect current clinical practices.

## 5 CONCLUSION

In conclusion, AGNHW emerges as a potent therapeutic agent with a broad spectrum of action. Its capabilities span from bolstering neurological outcomes refining immune cell dynamics, to modulating pivotal signaling pathways, making it a promising candidate for comprehensive ischemic stroke therapy.

## FUNDING INFORMATION

This study was partially supported by the funding of Beijing Tong Ren Tang Chinese Medicine Company Limited (Hong Kong, China).

## CONFLICT OF INTEREST STATEMENT

The authors declare no conflicts of interest.

## Supporting information


Data S1.

## Data Availability

The data that support the findings of this study are available on request from the corresponding author. The data are not publicly available due to privacy or ethical restrictions.
