# Supplementary material for: Comprehensive immune modulation mechanisms of Angong Niuhuang Wan in ischemic stroke: Insights from mass cytometry analysis
Source: CNS Neurosci Ther. 2024 Jul 29;30(7):e14849. doi: 10.1111/cns.14849 (PMC11286541; doi:10.1111/cns.14849)
Supplement: Supplementary file 1 — Data S1. [file CNS-30-e14849-s001.pdf]

Supplementary Figure 1

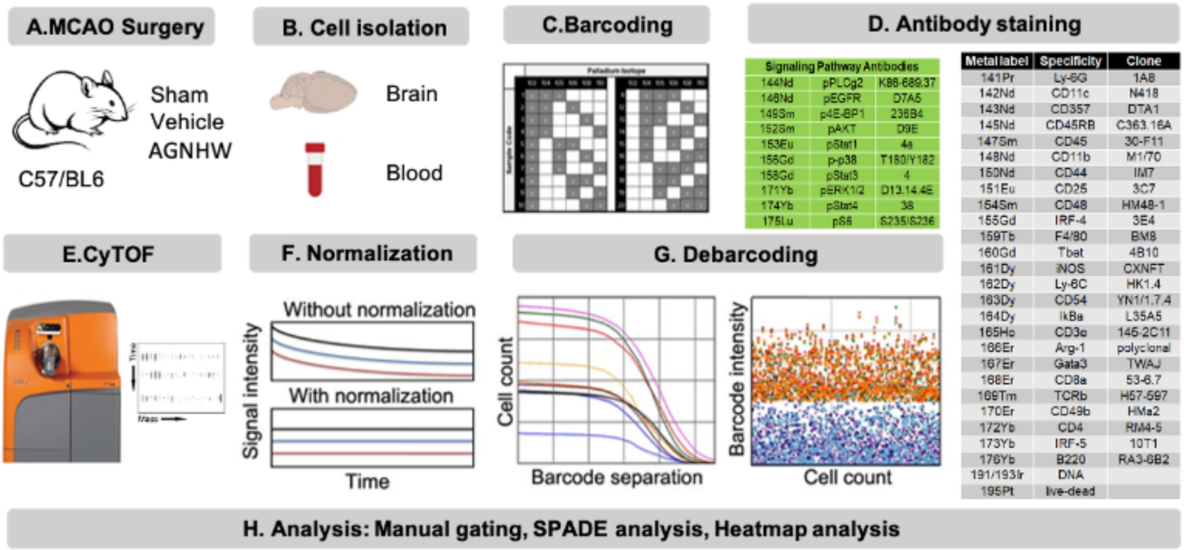

Figure S1. Experimental workflow. Mice were euthanized for tissue collection. The ischemic brain hemisphere and peripheral blood were collected at 3d after brain ischemia and immune cells were isolated and barcoded by a combination of three palladium (Pd) mass tags. Cells from the same tissue types were collected at the same time points, pooled, and stained using metal-labeled 35 antibodies. The CyTOF data was analyzed using Cytobank and presented by viSNE and standard dot pots. The marker intensity was analyzed using a heatmap. The cell number correlations were analyzed using the R programming and presented as the network.

Supplementary Figure 2

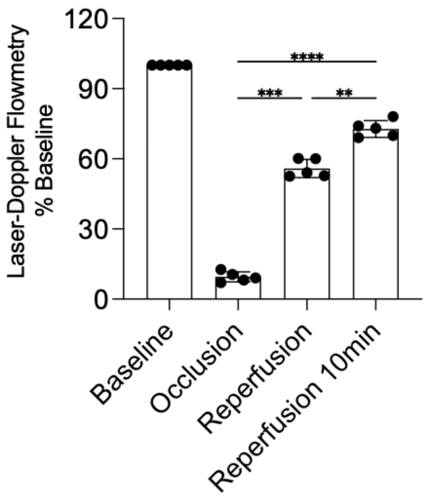

Figure S2. Regional cerebral blood flow (rCBF) was measured using a LASERFLO BPM^2 blood perfusion monitor. Laser Doppler Flowmetry values were recorded before surgery

(baseline), immediately after the filament was inserted in MCA, shortly after the reperfusion and 5 minutes after the reperfusion. Data are presented as Mean±SEM, n=5 per group. *P<0.05, respectively, between the two indicated groups.

Supplementary Table 1

RT-PCR Primer sequences

	Primer Sequence	
Gene	Forward	Reverse
GAPDH	GCCAAGGCTGTGGGCAAGGT;	TCTCCAGGCGGCACGCAGA
M1		
iNOS	GGTGAAGGGACTGAGCTGTT;	ACGTTCGTTCTCTTGCA
IL-1β	ACGCTTACCATGT GAGCTG;	GCCACAGGGATTTTGTCGTT
MCP-1	ACGCTTCTGGGCCTGTTGTT;	CCTGCTGCTGGTGATTCTCT
CCL-2	TAGCATCGTGCTGTCTC;	CAGCCGACTCATTGGGATCA
TNF-α	TCGGTCCCAACAAGGAGGAG;	GGGTTGTCACTCGAGTTTTG
M2		
CD206	CAAGGAAGGTTGGCATTTGT;	CCTTTCAGTCCTTTGCAAGC
Arg-1	TCACCTGAGCTTTGATGTCG;	CTGAAAGGAGCCCTGTCTTG
YM-1	CGAGGTAATGAGTGGGTTGG;	CACGGCACCTCCTAAATTGT
IL-10	AAATAAGAGCAAGGCAGTGG;	GTCCAGCAGACTCAATACACA
TGF-β	TGCGCTTGCAGAGATTAAAA;	CGTCAAAAGACAGCCACTCA

Supplementary Table 2

CyTOF antibody panel. The full metal-conjugated antibody panel used for CyTOF experiments. The amount of each antibody is referred to one sample with $3*10^6$ cells, at a 100µl final staining volume.

Isotope tag	Antibody	Host	Clone	Company
141Pr	Ly6G	Mouse	1A8	Fluidigm
142Nd	CD11c	Mouse	N418	Fluidigm
143Nd	CD357	Mou	DTA1	Fluidigm
145Nd	CD45RB	Mouse	C363.16A	Fluidigm
147Sm	CD45	Mouse	30-F11	Fluidigm
148Nd	CD11b	Mouse	M1/70	DVS Science
150Nd	CD44	Human/Mouse	IM7	DVS Science
151Eu	CD25	Mouse	3C7	Fluidigm
154Sm	CD48	Mouse	HM48-1	DVS Science
159Tb	F4/80	Mouse	BM8	DVS Science
162Dy	Ly6C	Mouse	HK1.4	Fluidigm
163Dy	CD54	Mouse	YN1/1.7.4	Fluidigm
165Ho	CD3e	Mouse	145-2C11	DVS Science
168Er	CD8a	Mouse	53-6.7	Fluidigm
169Tm	TCRβ	Mouse	H57-597	Fluidigm
170Er	CD49b	Mouse	HMa2	DVS Science
172Yb	CD4	Mouse	RM4-5	Fluidigm
176Yb	CD45R/B220	Human/Mouse	RA3-682	DVS Science
144Nd	pPLCg2	Mouse	K86-689.37	DVS Science
171Yb	pERK1/2	Mouse	D13,14,4E	Fluidigm
146Nd	pEGFR	Mouse	D7A5	Fluidigm
156Gd	p-p38	Mouse	T180/Y182	Fluidigm
153Eu	pStat1	Mouse	4a	Fluidigm
174Yb	pStat4	Mouse	38	DVS Science
158Gd	pStat3	Mouse	4	Fluidigm
152Sm	pAKT	Mouse	D9E	Fluidigm
149Sm	p4E-BP1	Mouse	23684	Fluidigm
175Lu	pS6	Mouse	S235/S236	DVS Science
155Gd	IRF-4	Human/Mouse	3E4	Fluidigm
173Yb	IRF-5	Mouse	10T1	Fluidigm
161Dy	iNOS	Mouse	CXNFT	Fluidigm
166Er	Arg-1	Mouse	Polyclonal	Fluidigm
160Gd	Tbet	Human/Mouse	4B10	Fluidigm
167Er	Gata3	Human/Mouse	TWAJ	Fluidigm
164Dy	IκBa	Mouse	L35A5	Fluidigm
